# Case Report: Autoimmune Encephalitis Associated With Anti-glutamic Acid Decarboxylase Antibodies: A Pediatric Case Series

**DOI:** 10.3389/fneur.2021.641024

**Published:** 2021-04-12

**Authors:** Changhong Ren, Haitao Ren, Xiaotun Ren, Weihua Zhang, Jiuwei Li, Lifang Dai, Hongzhi Guan, Fang Fang

**Affiliations:** ^1^Department of Neurology, National Center for Children's Health, Beijing Children's Hospital, Capital Medical University, Beijing, China; ^2^Department of Neurology, Peking Union Medical College Hospital, Chinese Academy of Medical Sciences and Peking Union Medical College, Beijing, China

**Keywords:** anti-glutamic acid decarboxylase 65, pediatric, autoimmune encephalitis, extralimbic encephalitis, limbic encephalitis

## Abstract

**Background:** Antibodies against glutamic acid decarboxylase (GAD) are associated with various neurologic conditions described in patients, including stiff person syndrome, cerebellar ataxia, refractory epilepsy, and limbic and extralimbic encephalitis. There have been some case reports and investigations regarding anti-GAD65 antibody-associated encephalitis in adult populations, but pediatric cases are rare. We retrospectively analyzed the clinical data of three anti-GAD65 antibody-positive patients to explore the diversity and clinical features of anti-GAD65 antibody-associated pediatric autoimmune encephalitis.

**Methods:** The clinical data of a series of three patients positive for anti-GAD65 antibody were retrospectively analyzed. GAD65 antibodies were determined in serum and CSF using a cell-based assay.

**Results:** All three patients were female, and the onset ages were 4 years and 9 months, 6 years, and 16 years old. Their clinical phenotypes included autoimmune limbic encephalitis, extralimbic encephalitis, and encephalitis combining limbic and extralimbic encephalitis. The clinical symptoms included seizures, memory deficits, drowsiness, dysautonomia, and headache. All patients had abnormal carinal MRI and EEG. All patients received immunotherapy and had transiently good responsiveness, but one patient then experienced relapse. In follow-up, one patient with extralimbic encephalitis recovered completely, while two patients with limbic involvement had poor outcomes with refractory focal epilepsy.

**Conclusion:** In addition to limbic encephalitis, extralimbic encephalitis is also an important phenotype in patients who are positive for anti-GAD65 antibodies. Early diagnosis and immunotherapy can improve the symptoms. However, patients with limbic encephalitis often have refractory epilepsy in the chronic phase and have a poor long-term outcome.

## Introduction

Glutamic acid decarboxylase (GAD), an enzyme that converts the excitatory neurotransmitter glutamic acid to the inhibitory neurotransmitter γ-aminobutyric acid (GABA), is widely distributed within the pancreas, nervous system, and other organs (1). Anti-GAD65 antibodies (GAD65-Abs) have been described as biomarkers not only for type 1 diabetes mellitus but also for several neurological syndromes, such as, stiff-person syndrome (SPS), cerebellar ataxia (CA), limbic encephalitis (LE), extralimbic encephalitis (ELE), and drug-resistant epilepsy (EP) (2–4). There have been some case reports and investigations regarding GAD65-Ab-associated encephalitis in adult populations, but pediatric cases were rare, and no cases involving Chinese children had been reported (5–11). Here, we report the first case series of GAD65-Ab-associated encephalitis in Chinese children.

## Materials and Methods

### Study Population

This study was performed in 180 pediatric patients (onset age <18 y) with possible autoimmune encephalitis from January 2018 to January 2020 who were admitted to the Department of Neurology, Beijing Children's Hospital. Serum and CSF samples were obtained for the detection of autoantibodies, including autoimmune encephalitis antibody profiles (NMDAR, LGI1, GABAR, CASPR2, AMPA1, AMPA2, DPPX) and paraneoplastic antibody profiles (Hu, Yo, Ri, Cv2, Ma2, and amphiphysin). Patients who had positive anti-GAD65 antibody in paired serum and CSF samples and had clear evidence of intrathecal synthesis (IS) were included in this study. Clinical and neuroimaging data were collected, and regular follow-ups were conducted. All patients or their guardians provided written informed consent for the clinical assessment, and the study was registered with the Registration Project for Pediatric Autoimmune Encephalitis (medical ethics committee approval number 2019-K-300).

### Detection of Anti-GAD65 Antibodies and Other Autoantibodies

Anti-GAD65 was detected in paired serum and cerebrospinal fluid (CSF) samples at the Neurological Laboratory of Peking Union Medical College Hospital by using a cell-based assay (CBA, EUROIMMUN Neurology Mosaic 13 A, FA 112d-1005-13 LS). Tissue-based assay (TBA) and ELISA were used to confirm the results.

The test steps were performed, as recommended in the kit, as follows: 30 μl of the serum or plasma (dilution fold 1:10) or cerebrospinal fluid (original sample) was added to the reaction zones of the sample plate, and slides containing thin slices of the biological substrates were then placed onto the plate. The slides were incubated for 30 min at room temperature, flushed with phosphate buffer solution, and incubated with 25 μl of sheep anti-IgG labeled with FITC for 30 min at room temperature. They were then rinsed with PBS and observed under a fluorescence microscope.

## Results

### Clinical Characteristics

Three of 180 pediatric patients tested positive for anti-GAD65 Ab in both serum and CSF samples. The clinical data are summarized in Table 1.

**Table 1:** Pediatric cases of anti-GAD65 antibody-associated encephalitis in our study and previous studies.

**Case**	**Gender/onset age (year)**	**Clinical features**	**Autoimmune disorders**	**GAD65 antibody titer**		**OCB**	**Brain MRI**	**EEG**	**Immunomodulatory** **treatment**	**Prognosis**
				**Serum**	**CSF**					
P1	F/6	Seizure, headache, memory deficient	None	1:100 (CBA)	1:320 (CBA)	Positive	Bilateral hippocampus	Right-sided epileptiform discharges	IVIG, MP, oral steroid	Refractory focal seizures
P2	F/16	Seizure, memory deficient, depression, dysautonomia	Thyroiditis	1:32 (CBA)	1:32 (CBA)	Positive	Initial: bilateral hippocampal 15th month: bilateral frontal lobe, left parietal lobe, right temporal lobe and insular cortex and subcortical white matter and bilateral hippocampus 5 years follow-up: parenchymal atrophy	Slowed theta rhythm with bilateral temporal spike-wave discharges	IVIG, MP, oral steroid, RTX	Persistent memory impairment and refractory focal seizures
P3	F/4y9 m	Vomiting, headache, confusion	None	1:100 (CBA)	1:320 (CBA)	Positive	Brainstem, thalamus, basal ganglia, bilateral cerebral, and cerebellar hemispheres	Slowed theta rhythm	IVIG, MP, oral steroid	Complete recovery
P1′ [5]	M/6	Epilepsia partialis continua, aphasia	Type 1 Diabetes Mellitus	19610 U/ml (normal range <1.0 U/ml)	3325 U/ml (normal range <1.0 U/ml)	NM	Initially: normal Later: lesions of the gray matter involving the occipital and frontal cortex, left insular region and cerebellum	Left-sided epileptiform discharges and slowing	High-dose steroids, IVIG, PE	Seizure free
P2′ [6]	F/16	Complex partial seizure and status epilepticus, academic function decline	Common variable immune deficiency (CVID)	>300 IU/ml (normal range 0–1.45)	>300 IU/ml (normal range 0–1.45)	NM	Initially: bilateral hippocampal T2 hyperintensities 1-year later: bilateral mesial temporal sclerosis	Bilateral temporal onset complex partial status epilepticus	MP, IVIG	Refractory seizures
P3′ [7]	F/2.1	Refractory seizures, developmental regression, memory impaired, ataxia	Type 1 Diabetes mellitus	3400 IU/ml (normal range <10 IU/ml)	13 U/ml (normal range <1 U/ml)	Positive	Initially: normal 3 years later: bilateral hippocampal, cortical, and cerebellar atrophy	Multifocal discharges and right frontal seizures	MP, PE, MMF, RTX	Clinical improvement but had refractory seizures
P4′ [8]	M/15	Headache, transient memory disturbance, seizures, behavior change	None	1:160000 (CBA)	1:128000(CBA)	Positive	T2/FLAIR signal of the right hippocampus and amygdala, mildly increased signal in the left amygdala	Interictal epileptiform discharges arising independently from right frontotemporal and left posterior head regions	IVIG, RTX, prednisone	Excellent seizure control; improvement of transient global amnesia-like episodes
P5′ [9]	M/7	Behavioral changes, dysphagia, ptosis, diplopia, anddrowsiness	None	Positive	Positive	NM	Normal	Epileptiform abnormalities both temporal regions	IVIG, PE	Complete recovery
P6′ [10]	F/9	Temporal lobe seizures, generalized tonic-clonic seizures, Mood and behavioral disturbances, autonomic imbalance	None	Positive	Positive	NM	Normal	Slowed theta rhythm with bilateral fronto-temporal spike wave discharges	MP, IVIG, RTX	6 months: died

*P, patients in our study; P, patients in a previous study; OCB, oligoclonal band; RTX, rituximab; MP, methylprednisolone; PE, plasma exchange; IVIG, intravenous immunoglobulin; MMF, mycophenolate mofetil; CSF, cerebrospinal fluid; CBA, cell-based assay; MRI, magnetic resonance imaging; NM, not mentioned*.

### Case 1

**Figure 1:**
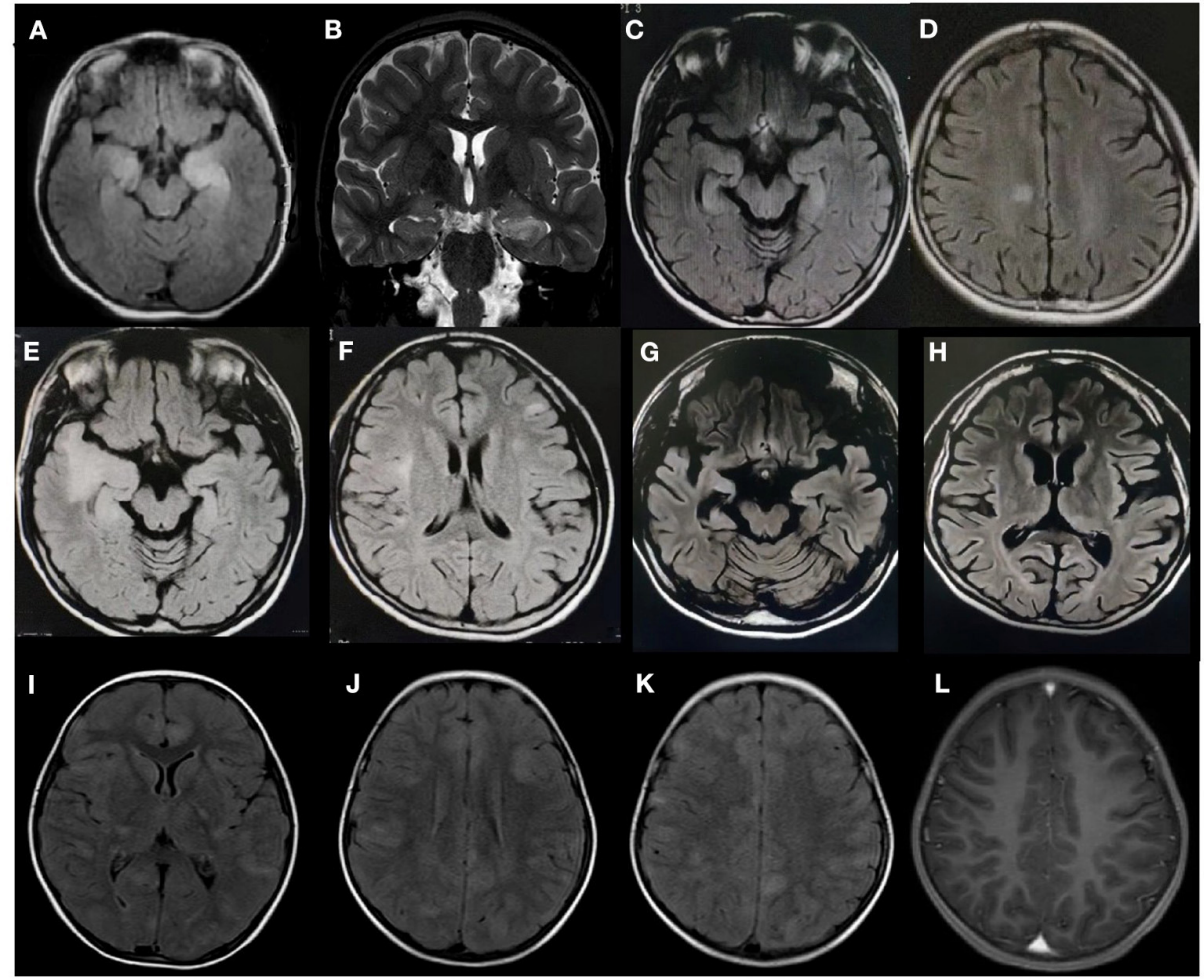
Brain magnetic resonance imaging findings of three patients with anti-glutamic acid decarboxylase 65 (GAD65) antibody-associated neurological syndromes. Patient 1: **(A)** An axial fluid-attenuated inversion recovery (FLAIR) sequence and **(B)** a coronal T2 sequence show increased signals in the bilateral hippocampus. Patient 2: **(C,D)** Axial FLAIR sequences demonstrate hyperintensity in a focal area of the right cingulate cortex and the bilateral hippocampus upon initial presentation, **(E,F)** new cortical/subcortical lesions at 15 months, and **(G,H)** progressive atrophy and temporal sclerosis at the 5-year follow-up. Patient 3: **(I–K)** Axial FLAIR sequences demonstrate diffuse abnormalities in the brainstem, thalamus, basal ganglia, and bilateral cerebral hemispheres; **(L)** Enhanced MRI shows no enhancement.

A 6-year-old girl presented with a 3-month history of intermittent focal seizures, headache, and decreased memory function. Her mother had hyperthyroidism, which was controlled very well with drugs. Her neurological examination was unremarkable. Routine biological tests, including biochemical tests and serum screening tests for infectious disease, metabolic abnormalities, systemic autoimmune diseases, tumor markers, and thyroid function, were unremarkable. Brain MRI showed abnormally high T2 and fluid-attenuated inversion recovery (FLAIR) signals in the bilateral hippocampus in association with swelling (Figures 1A,B). A 2-h video-electroencephalography (VEEG) recording showed that the right hemisphere had epileptiform activity. CSF analysis revealed eight white blood cells per cubic millimeter, normal glucose and protein, and positive oligoclonal banding (OCB). Serum and CSF screening for paraneoplastic antibodies were negative, as was tumor screening. However, both serum and CSF were positive for GAD65-Abs and had titers of 1:100 and 1:320, respectively. The patient was treated with intravenous methylprednisolone (20 mg/kg.d × 3 d) and immunoglobulins (IVIG) (2 g/kg). Two weeks later, her headache and seizure had improved, and she was discharged. Eighteen months after diagnosis, although, taking three anti-epileptic drugs (levetiracetam 50 mg/kg.d, oxcarbazepine 40 mg/kg.d, and topiramate 5 mg/kg.d), the patient still had focal seizures one-two times every week. Repeated brain MRI showed that the abnormal signals improved.

### Case 2

A 16-year-old girl presented with a 2-year history of remitted focal seizures and memory deficits. She was diagnosed with thyroiditis at 11 years old, and her father had hyperthyroidism. Initial brain MRI revealed bilateral hippocampal hyperintensity in T2-weighted sequences and FLAIR sequences (Figures 1C,D). CSF analysis demonstrated pleocytosis (310 white blood cells per cubic millimeter), but the cytopathology was unremarkable. EEG revealed a slowed theta rhythm with bilateral temporal spike-wave discharges. The thyroid peroxidase (TPO) antibody titer was increased to 149.10 IU/ml (normal 0–60 IU/ml). Five months after onset, screening for autoimmune encephalitis-associated antibodies showed that the patient's serum and CSF were both positive for GAD65-Abs at a titer of 1:10 (TBA, by EUROIMMUN), OCB was positive, and the Ig index was increased to 1.34 (normal: 0.3–0.7). The patient's symptoms and memory were transiently improved after IVIG (2 g/kg) and methylprednisolone (20 mg/kg.d × 3 d) were given. Fifteen months after onset, she developed autonomic imbalance, including dysrhythmia, hypothermia/hyperthermia, and hyperhidrosis. Repeat brain MRI showed abnormalities in the bilateral frontal lobe, left parietal lobe, right temporal lobe, and insular cortex as well as the subcortical white matter and bilateral hippocampus (Figures 1E,F). Both serum and CSF were still positive for GAD65-Abs, with titers of 1:10 and 1:32, respectively. However, the symptoms of refractory focal motor seizures and dysautonomia did not respond to treatment with antiepileptic drugs, methylprednisolone, immunoglobulins, and one cycle of rituximab (375 mg/m^2^ × 4 times). Twenty-five months after onset, the patient developed persistent depression and was given sertraline. For economic reasons, she was irregularly receiving IVIG and steroid immunotherapy at the local hospital. At the last follow-up, 5 years after onset, she had persistent memory impairment and refractory focal seizures. Repeat brain MRI showed parenchymal atrophy (Figures 1G,H).

### Case 3

**Figure 2:**
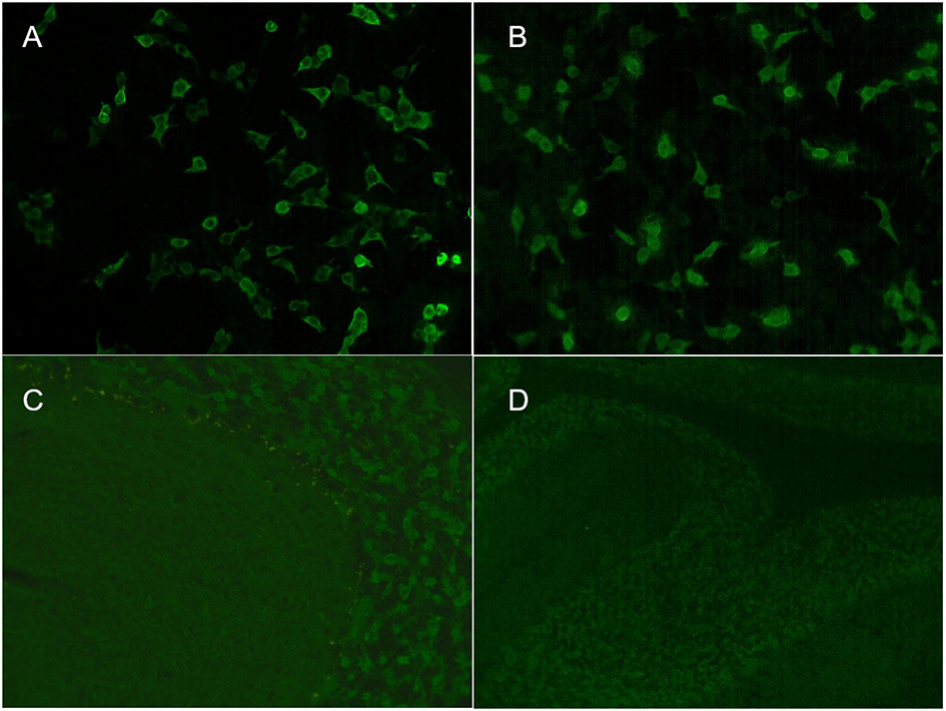
Indirect immunofluorescence assays of serum and cerebrospinal fluid (CSF) in patient 3. Cell-based assays of serum **(A)** and CSF **(B)** were both positive. Tissue-based assays of serum **(C)** and CSF **(D)** were both positive.

A girl aged 4 years and 9 months presented with a 1-day history of fever, vomiting, headache, and diplopia followed by coma. Her personal history and family history were unremarkable. Neurologic examination showed coma (Glasgow Coma Scale score 8), hyperactive deep tendon reflexes, and a positive Babinski sign. Brain MRI showed multiple diffuse abnormalities in the brainstem, thalamus, basal ganglia, and bilateral cerebral and cerebellar hemispheres on T2-weighted FLAIR imaging without enhancement (Figures 1I–L). A 2-h VEEG recording showed a slowed theta rhythm. CSF analysis revealed 11 white blood cells per cubic millimeter, along with normal glucose and protein. CSF cytopathology was unremarkable. Both serum and CSF were positive for GAD65-Abs, with titers of 1:100 and 1:320, respectively (Figure 2). OCB was positive. Screenings for paraneoplastic antibodies, anti-myelin oligodendrocyte glycoprotein (MOG) antibodies, and tumors were all negative. The patient was treated with methylprednisolone (20 mg/kg.d × 3 d) and IVIG (2 g/kg) and made a full recovery. After 2 weeks, she had no remaining symptoms and was discharged. A follow-up MRI after 1 month showed that the scope of the lesions was narrowed, the signal intensity was decreased, and the CSF GAD65-Ab titer was reduced to 1:32. At the last follow-up, 13 months after diagnosis, the patient had no symptoms, and MRI was normal.

### Antibody Detection Results

The serum antibody titers were 1:100, 1:10, and 1:32 in cases 1, 2, and 3, respectively. The CSF antibody titers were 1:320, 1:32, and 1:100 in cases 1, 2, and 3, respectively (Figure 2). All three patients had positive TBA in serum and CSF and serum concentrations >2,000 IU/ml by ELISA. All three patients tested negative for an autoimmune encephalitis antibody profile (NMDAR, LGI1, GABAR, CASPR2, AMPA1, AMPA2, DPPX) and a paraneoplastic antibody profile (Hu, Yo, Ri, Cv2, Ma2, and amphiphysin).

## Discussion

GAD65-Abs are a rare but important cause of pediatric autoimmune encephalitis. As an intracellular antibody, the pathogenic role of GAD65 antibody in neurologic disorders is unclear. However, patients with high antibody concentrations had well-defined clinical phenotypes that have been classically associated with GAD65- Abs, such as, SPS, CA, AE, and EP. Compared to adults, the most common neurologic disorders in children are LE, ELE, and EP.

Several pediatric cases have been reported in the literature (summarized in Table 1). Here, we report three pediatric patients with GAD65-Ab-associated AEs. Among the nine pediatric patients reported in the literature and in this article, five cases were LE, three cases were ELE, and one case was a combination of limbic and extralimbic encephalitis. The clinical symptoms included seizures (seven cases), memory loss (five cases), loss of consciousness (three cases), behavioral abnormalities (three cases), cognitive impairment (two cases), headache (two cases), autonomic symptoms (two cases), ataxia (one case), dysphagia (one case), and aphasia (one case). LE patients clinically present with subacute onset impaired working memory, psychiatric symptoms, and seizures. MRI shows bilateral T2-W/FLAIR hyperintensities that are highly restricted to the temporal lobes in the acute phase, and hippocampal sclerosis/atrophy often occurs during follow-up (12). ELE is also observed in patients with GAD-Abs. The clinical manifestations of these patients may resemble those of LE, or without seizures, they may have severe autonomic symptoms, brainstem symptoms, cerebellar symptoms and so on. MRI presents with hyperintense cortical/subcortical lesions in T2W/FLAIR MRI sequences and hypermetabolic lesions in PET scans (3), usually without contrast enhancement. Cases combining limbic and extralimbic encephalitis or normal MRI have also been described. Patient three in our study did not initially have seizures, and the clinical manifestations and MRI mimicked pediatric acute disseminated encephalomyelitis. Therefore, GAD65-Abs should be considered an important cause of extralimbic encephalitis and should be tested for patients with that condition. Patient two presented with dysautonomia, which was accompanied by depression, a symptom that is rarely reported. This suggests that psychological assessment may also be important in long-term follow-up.

The diagnosis of GAD65-Ab-associated neurological syndromes is based on clinical grounds plus the presence of high anti-GAD65 Ab titers or the detection of intrathecal synthesis (IS) in CSF (3). Although GAD65-Abs can be detected through a number of methods, the most common is ELISA in prior research. In our research, we detected GAD65-Abs in serum and CSF through CBA and used TBA and ELISA to confirm the positive cases. Maarten J. Titulaer (13) compared three different laboratory techniques in GAD65-Ab-associated syndromes, including ELISA, CBA, and immunohistochemistry, and found that 100% of high-concentration samples were positive for CBA, and 96% of low-concentration samples were negative. CBA is more specific, especially if a different antibody is concomitantly present in the tested sample. According to prior studies and our own case series, CBA could be considered a good screening method for suspected patients. In addition, TBA has an important value in defining antibodies. The specific affinity of CSF and serum samples with rat brain tissue is an indirect verification of antigenicity. For CBA-positive patients, TBA positivity was further verified. Especially for patients with negative CBA, the significance of positive TBA is more important. All three patients in our study showed high titers of anti-GAD65 antibodies in serum and cerebrospinal fluid, and the antibody titer reduced as clinically improved, but did not disappear. Both patients one and two had refractory epilepsy in the chronic phase. The antibody titer of patient one was significantly higher than that of patient two, but the frequency and severity of seizures were lower than those of patient two. This also confirms that the titers of anti-GAD65 antibodies seem to be not associated with the frequency of seizures and the severity of disease among different individuals. However, reduced antibody titers are observed in patients who clinically respond to treatment, but persistently high titers are often associated with poor clinical response.

There are no prospective trials to guide management, so the treatment experiences are all derived from case reports. In general, this syndrome is considered to be less responsive to immunotherapy than other subsets of autoimmune encephalitis in which antibodies target cell surface proteins. The first-line treatment is intravenous methylprednisolone, intravenous immunoglobulin (IVIG), and plasma exchange, the same as in other autoimmune encephalitis. Clinically, some patients had a transient good responsiveness to first-line treatment with a significant decrease in seizure frequency and improvement in memory and behavioral disturbances. Some patients may require second-line therapy, including rituximab (RTX), cyclophosphamide (CPM), or mycophenolate mofetil (MMF) (14), but the efficacy varies in different case reports. All nine reported pediatric patients (including patients reported in the literature and in this article) had a transient good response after first-line immunotherapy. However, after 6 months ~6 years of follow-up, three ELE patients had good clinical recovery without residual MRI lesions, and one LE patient died. The prognosis of the other five patients with limbic lobe involvement was poor; even after second-line therapy, all had refractory focal epilepsy and cognitive impairment, and four of them had hippocampal sclerosis/atrophy during follow-up. We think that limbic lobe involvement may be a risk factor for chronic refractory epilepsy and poor prognosis, but the sample size is small, the specific factors affecting the prognosis are currently unclear, and a large sample of research is still needed.

## Data Availability Statement

The raw data supporting the conclusions of this article will be made available by the authors, without undue reservation.

## Ethics Statement

The studies involving human participants were reviewed and approved by Registration Project for Pediatric Autoimmune Encephalitis (medical ethics committee approval number 2019-K-300). Written informed consent to participate in this study was provided by the participants' legal guardian/next of kin.

## Author Contributions

CR and HR participated in writing the manuscript. XR, WZ, JL, and LD participated in collecting the information of the paper and analyzing or interpreting the data. HG and FF participated in revising the manuscript. All authors contributed to the article and approved the submitted version.

## Conflict of Interest

The authors declare that the research was conducted in the absence of any commercial or financial relationships that could be construed as a potential conflict of interest.
